# SPECS: a non-parametric method to identify tissue-specific molecular features for unbalanced sample groups

**DOI:** 10.1186/s12859-020-3407-z

**Published:** 2020-02-17

**Authors:** Celine Everaert, Pieter-Jan Volders, Annelien Morlion, Olivier Thas, Pieter Mestdagh

**Affiliations:** 1 Center for Medical Genetics, Department of Biomolecular Medicine, Ghent University, Ghent, Belgium; 2 Cancer Research Institute Ghent, Ghent, Belgium; 3 Flemish Institute for Biotechnology, Ghent, Belgium; 4 I-Biostat, Data Science Institute, Hasselt University, Hasselt, Belgium; 5 National Institute for Applied Statistics Australia (NIASRA), University of Wollongong, Wollongong, Australia; 6 Department of Data Analysis and Mathematical Modelling, Faculty of Bioscience Engineering, Ghent University, Ghent, Belgium

**Keywords:** Specificity scoring, RNA-sequencing, GTEx

## Abstract

**Background:**

To understand biology and differences among various tissues or cell types, one typically searches for molecular features that display characteristic abundance patterns. Several specificity metrics have been introduced to identify tissue-specific molecular features, but these either require an equal number of replicates per tissue or they can’t handle replicates at all.

**Results:**

We describe a non-parametric specificity score that is compatible with unequal sample group sizes. To demonstrate its usefulness, the specificity score was calculated on all GTEx samples, detecting known and novel tissue-specific genes. A webtool was developed to browse these results for genes or tissues of interest. An example python implementation of SPECS is available at https://github.com/celineeveraert/SPECS. The precalculated SPECS results on the GTEx data are available through a user-friendly browser at specs.cmgg.be.

**Conclusions:**

SPECS is a non-parametric method that identifies known and novel specific-expressed genes. In addition, SPECS could be adopted for other features and applications.

## Background

To understand biology and differences among various tissues or cell types, one typically searches for molecular features (i.e. RNA, protein, metabolites) that display characteristic abundance patterns. In the most extreme case, these features display tissue- or cell-type restricted abundance profiles. Such specific features can provide insights in functional, development or disease mechanisms [1] or serve as biomarkers [2, 3]. Various consortium-based efforts have generated vast amounts of molecular data that can be exploited for this purpose. The Genotype-Tissue Expression (GTEx) project (https://gtexportal.org) and The Cancer Genome Atlas (TCGA) (https://www.cancer.gov/tcga) are examples of such rich resources containing RNA-sequencing based molecular features for thousands of samples derived from various individuals and tissue types [4]. To identify tissue-specific molecular features, several specificity metrics have been introduced, but these can suffer from data loss introduced by the requirement to collapse data from biological replicates. By introducing summary statistics, replicate data points are typically reduced to a single value (mean) or two values (mean and standard deviation). Examples of such metrics are Tau [5], the z-score [6], the Gini coefficient [7] and the tissue specificity index (TSI) [8]. Metrics that can handle biological replicates (e.g. JSD [9]) require equal sample sizes. Specificity metrics also differ in the output that is generated. Some (Tau, Gini, TSI) generate a single score often representing fold-changes between the mean values while others (z-score, JSD) generate a score per tissue. A thorough benchmark was performed for these scores, identifying Tau as the best overall method [10]. This benchmark focused on robustness of the scores by subsampling the tissues. Biological signal was evaluated by calculating the conservation of tissue specificity between mouse and human orthologs, and by assessing several tissue specific GO terms.

In this application note, we describe a novel non-parametric specificity score that is compatible with unequal sample group sizes, uses all individual datapoints and enables the detection of features that are specifically present or absent in one or more tissue types. We benchmarked our SPECS score with others by artificially introducing expression specificity in a large and heterogeneous RNA-sequencing data set.

## Results

The evaluation of individual features for predicting a binary biological status can be based on an estimate of the area under the ROC curve (AUC). The AUC can be either interpreted as an integrated performance metric of sensitivities and specificities over all possible thresholds, or as a measure for the overlap between the distributions of the feature in the two biological status groups. When more than two groups are present, and the aim is to evaluate a feature, in our case gene expression, for distinguishing one group from the others, we propose a new AUC-type of statistic (SPECS). This method still has the interpretation of a measurement for the overlap between two distributions (one group as compared to the pooled group of the others). Perfect non-overlapping abundance distributions where the distribution of the specific group is shifted to higher abundance values have a SPECS score of one. On the other hand, when the feature is absent in one group, the distribution of this group shifts to zero or lower abundance values and this results in a score of zero. On top, the method can be adjusted to account for the prevalence of the biological statuses in the target population (and hence correct for the group sample sizes in the available dataset).

Although features can be selected based on their ranking in terms of their estimated AUCs, this procedure is at risk for selection bias, i.e. a large estimated AUC may result from a feature with only a moderate AUC but with a large estimation variance. Efron et al. discussed this issue in detail [11], but did not apply this to the AUC. He proposed to correct the estimates by means of an empirical Bayes procedure, which can be seen as a Bayesian procedure for which no prior distribution need to be specified. As an advantage, Bayesian methods are known to be insensitive to selection bias. His formula is also known as Tweedie’s formula, described into more detail in Supplemental Methods 1.

**Fig. 1:**
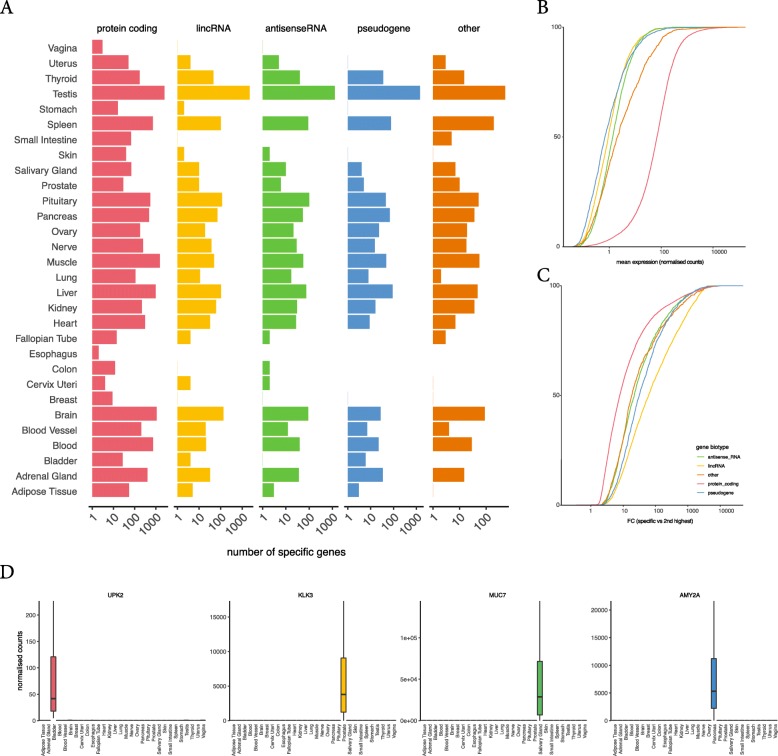
Known and novel genes are detected as specific for various biotypes. **a** The number of specific genes for each GTEx tissue and biotype shows that most specific genes are protein-coding. **b** Cumulative distribution of the mean expression of specific genes, shows that specific protein-coding genes are higher expressed compared to the other biotypes. **c** Cumulative distribution of the fold changes of specific genes and the 2nd tissue shows larger differences for lincRNA genes compared to other biotypes. **d** Examples of well-known specific genes; UPK2 for bladder, KLK3 for prostate, MUC7 for adrenal gland and AMY2A for pancreas

To evaluate our SPECS method, we made use of RNA-sequencing data from the GTEx (version 7) project [4] consisting of 12,766 samples belonging to 31 different tissues (7 to 1854 samples per tissue). We calculated the SPECS specificity score on normalized counts for all Ensembl (GRCh38.v85) genes (*n* = 56,202) using all samples. For 30 of the 31 tissues, 2 (esophagus) to 7948 (testis) specifically expressed genes were identified. Most of these genes are protein coding (*n* = 10,959), followed by lincRNAs (*n* = 3080), antisense genes (*n* = 2022) and pseudogenes (*n* = 1976) (Fig. 1a, Supplemental Figure 1 and Supplemental Table 1). In addition, the method has the ability to identify genes that are highly specific for two (or more) tissues, with specificity scores that are slightly lower. As expected, the tissues with the highest number of common specific genes are biologically related such as spleen and blood, or brain and pituitary or muscle and hart.

Besides genes that are specifically abundant in a tissue, our method also enables the identification of genes that are specifically repressed in a given tissue. These so-called disallowance genes [12] were found for 17 tissues ranging from 2 (salivary gland) to 1989 (blood) genes. Most of these are protein coding genes (Supplemental Figure 2). Distributions of the SPECS score are highly similar for all tissue types, except for testis (which is known to be enriched for tissue-specific genes). Most genes have a SPECS score around 0.5 (Supplemental Figure 3). For all specifically abundant genes we calculated fold changes between the specific tissue(s) and all other tissues. The fold changes for lincRNAs were typically higher than for other biotypes, in line with previous studies in which lincRNAs were shown to be more specific compared to protein coding genes [9] (Fig. 1b and c). The SPECS score is not impacted by abundance, however, measuring counts with RNA-seq is. Low abundant genes suffer from sampling bias and thus have a higher variance. In addition, zero counts can indicate real absence of gene expression or can occur when abundance falls below the detection threshold of the gene expression profiling method.

From our analyses, known specific genes are readily confirmed, such as kallikrein related peptidase 2 (KLK2) and 3 (KLK3, also known as PSA) for prostate, uroplakin 2 (UPK2) for bladder, mucin 7 (MUC7) for the salivary gland and amylase alpha 2A (AMY2A) for pancreas (Fig. 1d). For each tissue in GTEx, rank percentiles for the specific genes are pre-calculated and distilled into a web tool (specs.cmgg.be) where a user can select either their gene of interest to evaluate its specificity or a tissue of interest to identify the most specific genes.

To compare the SPECS score to other existing scores, we artificially introduced specificity in the GTEx expression dataset by multiplying true gene counts with a constant factor or by adding constant values to gene counts in one tissue type. To this purpose, a set of 1000 genes with small overall variabilities and a mean expression below 10 counts was selected (further referred to as the backgound set). For each experiment, counts for fifty randomly selected genes from the background set were manipulated as described above (see Methods for details) to introduce tissue-specificity. This process was performed independently for 5 random tissues. Since the different specificity scores each have their own scale and cut-off value, we used the ranks of the scores across all 1000 genes to compare methods. We expect our specificity-induced genes to rank high, and thus have low ranks. For each round of simulations, we then sum up these ranks for the specificity induced genes and compare these summed ranked values across all (five) simulations.

**Fig. 2:**
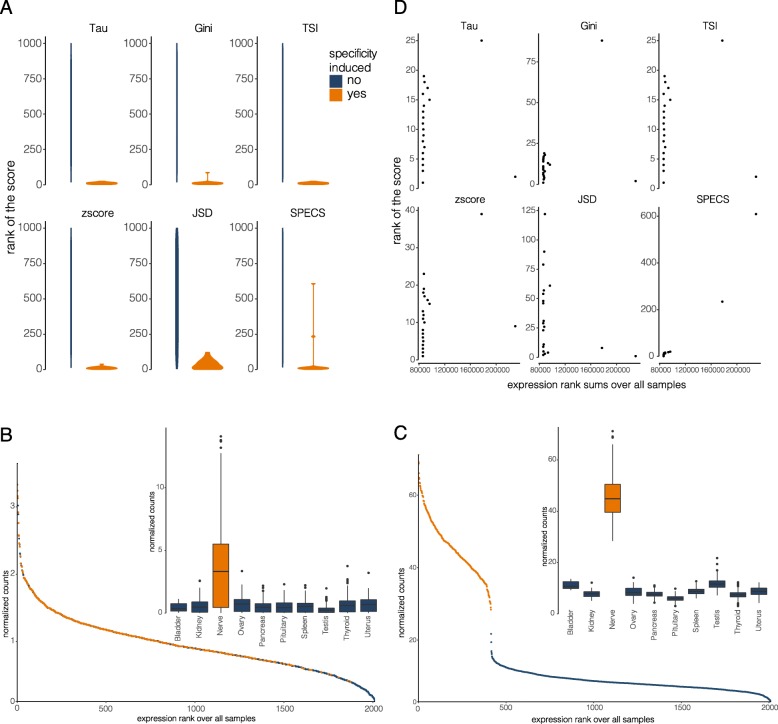
Benchmarking SPECS compared to the other scores by multiplication of the background signal in one tissue. **a** Ranked specificity score values for different metrics. Ranks are higher for SPECS compared to the other metrics. **b** A gene with induced specificity that is ranked higher SPECS compared to the other metrics shows a large expression overlap with the other tissues. **c** A gene with induced specificity, that is ranked lower all metrics shows less expression overlap with the other tissues. **d** Correlation between the summed rank of the gene expression with the rank of the score for each metric. SPECS shows the strongest correlation

No differences between the methods in the summed ranked values were observed for multiplication factors of 1, 2, 10 and 20 (respectively *p* = 0.914, *p* = 0.454, *p* = 106, *p* = 0.439). For multiplications with a factor of 3, 4, 5, 6 and 8, significant differences (*p* < 0.05) amongst specificity metrics were observed. When looking into this data (for a 5-fold multiplication), SPECS assigned lower scores, resulting in lower ranks, to some of the specificity induced genes (Fig. 2a) compared to the other methods. The expression profiles of these genes in the tissue with induced specificity were increased but showed clear overlap with the expression profiles in the other tissues (Fig. 2b), explaining the lower SPECS scores. Most other methods did not generate lower scores for these genes. To assess the impact of overlapping expression distributions on the specificity score, we first calculated a metric that reflects overlap. To this end, all samples were ranked based on expression of the gene, and the ranks of the samples belonging to the specificity induced tissue were summed. If the expression in the tissue with induced specificity is consistently higher, the rank sum in that tissue will be low (Fig. 2c). In contrast, if the expression of the specific tissue is overlapping the other tissue, the individual ranks will be higher, resulting in a higher rank sum (Fig. 2b). Plotting expression rank sums for all specificity induced genes versus the matching ranked scores of the metrics clearly shows that only SPECS has a reduced score when there is more expression overlap with other tissues (Fig. 2d), while this relationship is lacking for the other metrics.

**Fig. 3:**
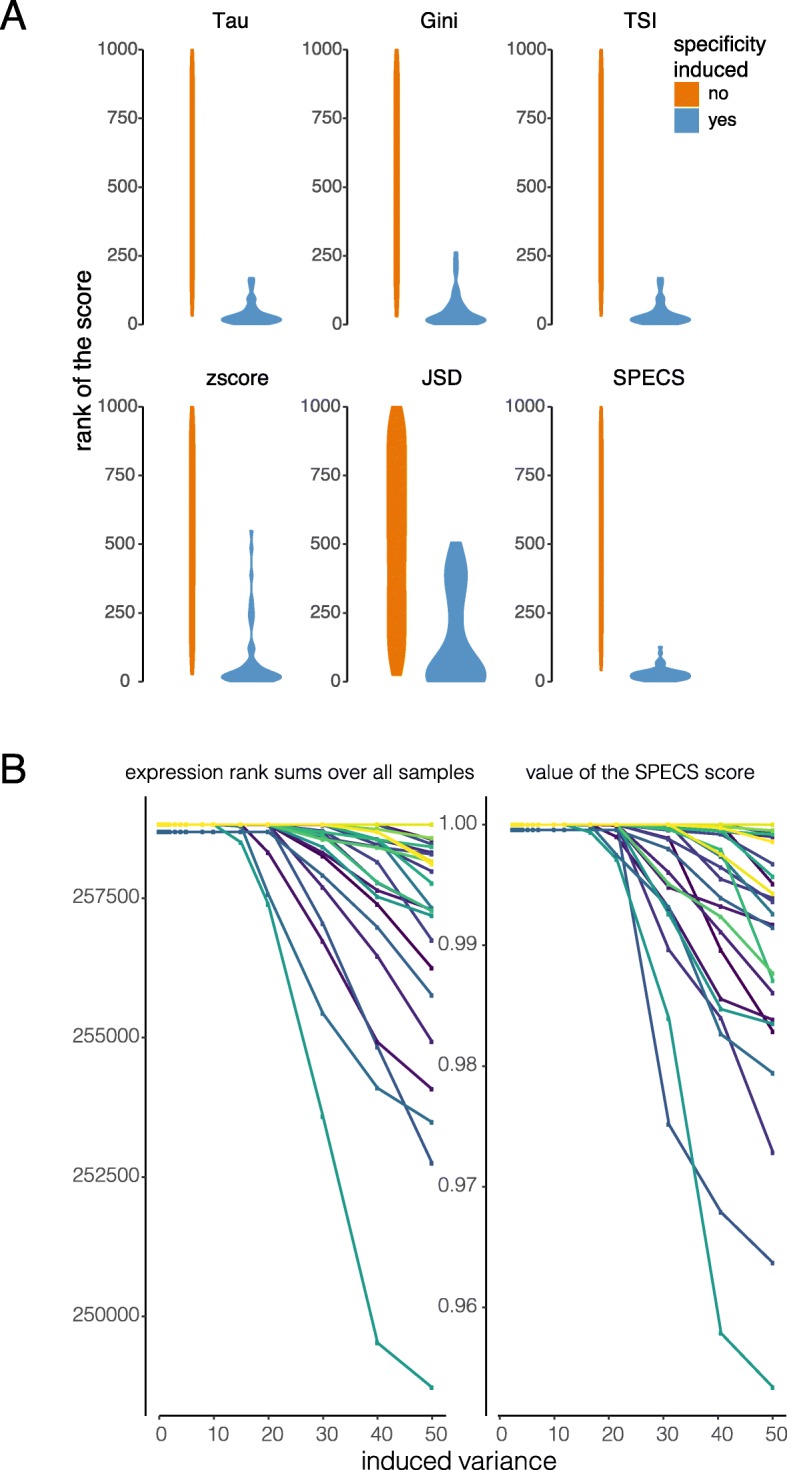
Benchmarking SPECS compared to the other scores by summation of a constant value to the background signal in one tissue. **a** Ranked score values of multiple metrics show higher ranks for SPECS compared to the other scores when adding 10 counts. **b** The impact of increasing variance on the SPECS score. Increasing variance results in increasing overlap of expression distributions, indicated by summed expression ranks. Each gene is represented by an individual line in the plot, colors indicate the same gene in each plot

To assess the impact of the variance, a constant count value was added to the gene count and multiple variance factors were introduced (see Methods for details). Compared to multiplying counts, adding a constant value excludes zero counts and maintains the variance. When adding 10, 15, 25, 50, 75, 100 and 1000 counts, clear differences between the performance of the methods are observed up to adding 50 counts (*p* < 0.0001). SPECS clearly outperforms the other methods for lower count values and is thus more sensitive to detect low abundant tissue-specific genes (Fig. 3a). To analyze the impact of the variance, we added 100 counts to the gene count and evaluated the specificity scores while increasing the variance (from original variance to 50 times higher variance). Specificity scores were insensitive to increasing variance for all metrics but SPECS (shown for 50 specificity induced genes in Supplemental Figure 4). This observation can be explained by the fact that SPECS is taking into account the variation. As expected, genes who’s SPECS scores decrease with increasing variance showed systematically more expression overlap with the other tissues (measured by the rank sum as explained higher) (Fig. 3b). For the genes that have a stable SPECS score the overlap is stable as well.

Additionally, we wanted to evaluate how SPECS deals with changing group sizes. Specificity was induced by randomly adding 100 counts to 50 genes from the background set and this for one tissue. From the specificity induced tissue samples, a random fraction of samples was subsampled (ranging between 20 and 100%) after which the SPECS score was calculated on this subsampled set. This was repeated five times for different randomly chosen tissues. No difference (*p* = 0.874) was observed for the SPECS score between the different group sizes. Finally, robustness was tested to show that SPECS is stable when using random fractions of the data. We therefore repeatedly (*n* = 5) subsampled equally sized sample groups (20% of the original data) from the original data. No changes in the SPECS score values for the specificity induced genes were observed (*p*-values between 0.158 and 0.411).

## Discussion

Current statistics to calculate specificity are collapsing datapoints within each label into a single value, whether or not with an additional variance metric, resulting in loss of information. Our non-parametric specificity score SPECS uses all data points to calculate a specificity metric. We calculated the score on the GTEx data and recovered known biology. We benchmarked SPECS with various established specificity scores and found that SPECS outperforms the other scores. SPECS is more sensitive to detect specific genes that are low abundant. Additionally, SPECS takes into account the variance, and therefore disfavors genes with overlapping expression distributions between tissues. In addition, SPECS is stable with changing sample sizes and robust. This specificity metric can be applied to any type of quantitative molecular data including protein expression or chip-sequencing. Not only tissues could be used as features but also cancer types or ethnic populations. Besides biological applications, we also see application potential in other fields such as economy and social sciences.

## Conclusion

SPECS is a non-parametric specificity score applicable on big data sets without data loss or reduction. In our example SPECS is shown to be useful to calculate tissue specific expression of genes, however, other applications are possible in molecular biology or beyond.

## Methods

Let the index *d* = 1,...,m_d_ refer to a particular sample state. Depending on the application and whether the user wants to give weight to a certain state, π_d_ is the prevalence of state *d* in the target population or π_d_ is equilibrated. Suppose there are m_g_ candidate features, i.e. g = 1,...,m_g_. Let Y_gd_ denote the outcome of feature *g* in state *d* with n_gd_ observations, so that the individual outcomes are denoted by Y_gdi_, i = 1, …,n_gd_. The Y_g-d_ notation denotes the outcome of feature *f* in all groups but the state *d*. The index *g* will be dropped in further notations. A feature is a characteristic for a given state if its outcome distribution for the given state shows no overlap with the outcome distributions of the other states. This means a larger AUC, given by:

1 $$ {p}_d=P\left\{{Y}_{-d}<{Y}_d\right\}=\sum \limits_{k\ne d}P\left\{{Y}_k<{Y}_d\right\}{\pi}_k $$

If p_d_ is close to zero or one, the distributions are well separated. The probabilities P{Y_k_ < Y_d_} are computationally fast to calculate. The probability P_kd_ = P{Y_k_ < Y_d_} is then estimated as:

$$ {\hat{P}}_{kd}=\frac{1}{n_k{n}_d}\sum \limits_{i=1}^{n_k}\sum \limits_{j=1}^{n_d}{I}_{ki;\mathrm{d}j} $$

with *I*_*ki*; *dj*_ a 0/1 indicator for the event *Y*_*ki*_ < *Y*_*dj*_.

Hence, an estimator of p_d_ is given by:

$$ {\hat{p}}_d=\sum \limits_{k\ne d}{\hat{P}}_{kd}{\pi}_k $$

Further selection of features can be performed based on the distributions of ${\hat{p}}_d$ as explained in Supplemental Methods 1. As this is a computationally intensive step for large data matrices, one can opt to select features based on a threshold. In our use case, we defined state-specific features as those where the score (${\hat{p}}_d$) for one state was above 0.95 and features that were specifically absent in one state as those with a score (${\hat{p}}_d$) lower than 0.05. If the score of 0.95 or 0.05 was reached in multiple states, the feature was defined as specific (present or absent) for all these states. The python implementation of the method is available at https://github.com /celineeveraert/SPECS.

To calculate SPECS, the count data was retrieved from the GTEXportal (www.gtexportal.org) and normalized by DESeq2 [13]. For the benchmarking, we selected 1000 low abundant (mean normalized counts between 0.1 and 10) and stable (lowest standard deviation between the tissue types) expressed genes to create a background set. We included samples from 10 tissue types with a variable sample number (11 to 490 samples per type). In this data set, we artificially introduced specificity and calculate various specificity metrics.

### Zscore [6]

$$ z=\frac{x_i-\mu }{\sigma } $$

μ is the mean of gene expression; σ is the standard deviation

### Gini coefficient [7]

$$ Gini=\frac{n+1}{n}-\frac{2{\sum}_{i=1}^n\left(n+1-i\right){x}_i}{n{\sum}_{i=1}^n{x}_i} $$

x_i_ has to be ordered starting at the smallest value

### Tau [5]

$$ \tau =\frac{\sum_{i=1}^n\left(1-{\hat{x}}_i\right)}{n-1};{\hat{x}}_i=\frac{x_i}{\underset{1\le i\le n}{\max}\left({x}_i\right)} $$

### TSI [8]

$$ TSI=\frac{\underset{1\le i\le n}{\max}\left({x}_i\right)}{\sum_{i=1}^n{x}_i} $$

### JSD Score [9]

$$ JS\left({p}^1,{p}^2\right)=H\left(\frac{p^1+{p}^2}{2}\right)-\frac{H\left({p}^1\right)+H\left({p}^2\right)}{2} $$

where H is the entropy of a discrete probability distribution:

$$ {\displaystyle \begin{array}{c}p=\left({p}^1,{p}^2..,{p}^n\right),0\le {p}_i\le 1\  and\ \sum \limits_{i=1}^n{p}_i=1\\ {}H(p)=-{\sum}_{i=1}^n{p}_i\ \log \left({p}_i\right)\end{array}} $$

The distance between two expression patterns (e) is defined as:

$$ JSD\left({e}_1,{e}_2\right)=\sqrt{JS\Big(}{e}_1,{e}_2\Big) $$

The tissue specificity for tissue t can then be defined as:

$$ \mathrm{JSD}\ \mathrm{Score}\ \left(\mathrm{e}|\mathrm{t}\right)=1-\mathrm{JSD}\left(e,{e}^t\right) $$

where *e*^*t*^ is a predefined expression pattern in which there is only expression in one tissue.

The tissue specificity score is than defined as the maximal score across all tissues.

To be able to compare these scores expressed on different scales, score ranks were used instead of the absolute score values. These score ranks are calculated over all genes (including the background set). As such, the need to define cut-off values was also avoided.

We define a specific gene as a gene that is systematically higher expressed compared to the background. We introduced higher expression by either multiplication of the counts or adding a constant number. If we multiply, zero counts remain zero and low counts can still appear in the background. To quantify the overlap, we calculated for each gene the expression-based rank of the sample over all samples, were 1 is the sample with the highest abundance. By summing these ranks, we have a proxy for systematically higher expression. A high rank sum indicates that some samples overlap with the non-specificity induced tissues which resulted in a higher rank across all samples and thus a higher rank sum.

To compare the metrics, ANOVA testing was used on five cycles of random specificity induction of 50 genes out of 1000 genes from the background set.

## Supplementary information

**Additional file 1.** Supplementary methods.

**Additional file 2.** Supplemental Figure 1. Heatmap of the median expression of the specific genes for each tissue, shows the degree of specificity. Supplemental Figure 2. Number of disallowance genes for each tissue and biotype is variable. Supplemental Figure 3. SPECS score distribuitions for all GTEx tissue types. Supplemental Figure 4. Most scores remain stable with a larger induced variance, while SPECS has a declining score.

**Additional file 3.** Supplementary Table SPECS results for all genes on GTEX.

## Data Availability

Code: https://github.com/celineeveraert/SPECS Precalculated GTEx data: https://specs.cmgg.be

## Abbreviations

AMY2A: Amylase alpha 2A
GTEx: Genotype-Tissue Expression
KLK2: Kallikrein related peptidase 2
KLK3: Kallikrein related peptidase 3
MUC7: Mucin 7
TCGA: The Cancer Genome Atlas
UPK2: Uroplakin 2
