# Supplementary material for: SPECS: a non-parametric method to identify tissue-specific molecular features for unbalanced sample groups
Source: BMC Bioinformatics. 2020 Feb 17;21:58. doi: 10.1186/s12859-020-3407-z (PMC7026976; doi:10.1186/s12859-020-3407-z)
Supplement: Supplementary file 2 — Additional file 2. Supplemental Figure 1. Heatmap of the median expression of the specific genes for each tissue, shows the degree of specificity. Supplemental Figure 2. Number of disallowance genes for each tissue and biotype is variable. Supplemental Figure 3. SPECS score distribuitions for all GTEx tissue types. Supplemental Figure 4. Most scores remain stable with a larger induced variance, while SPECS has a declining score. [file 12859_2020_3407_MOESM2_ESM.pdf]

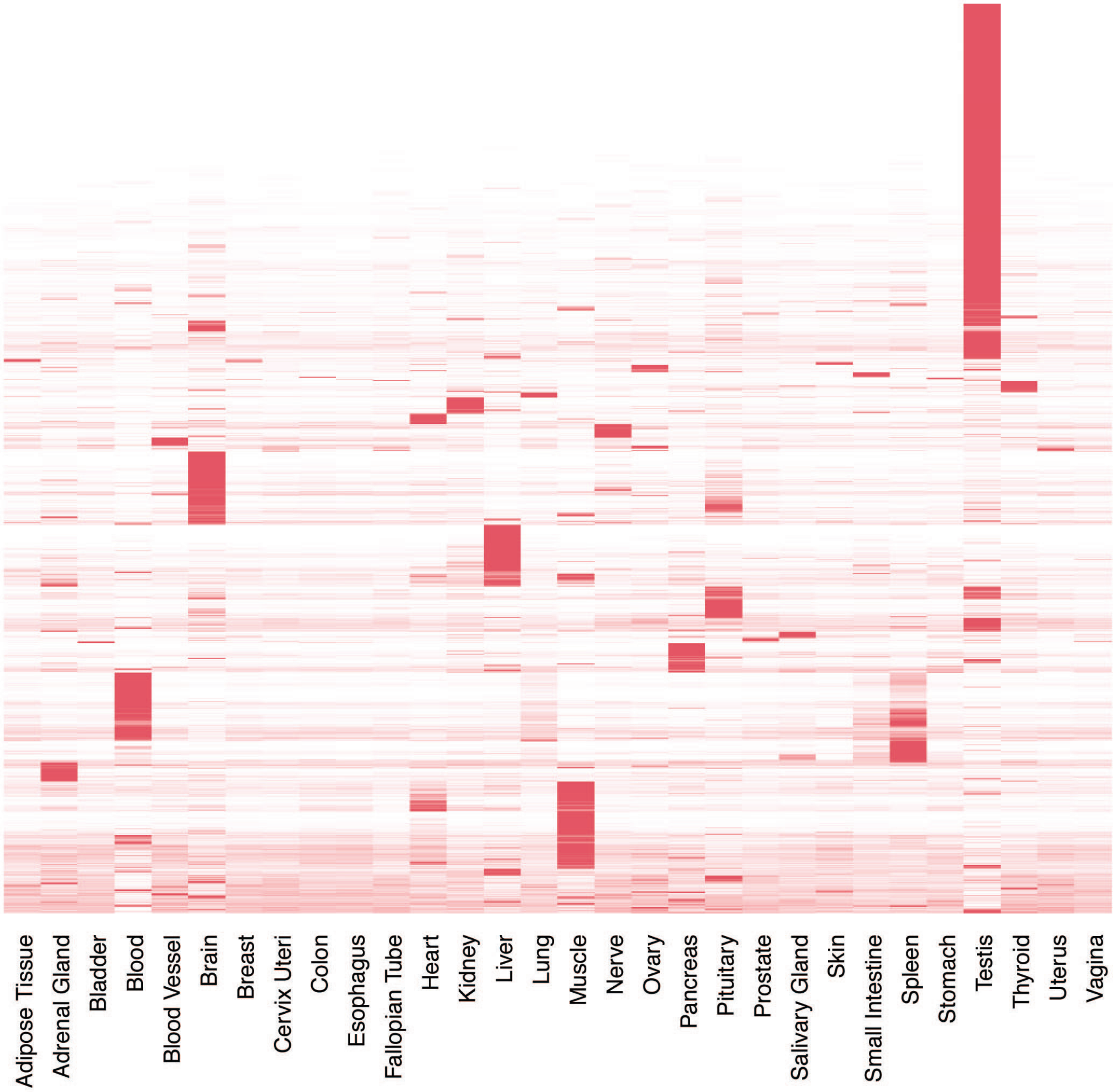

Supplemental Figure 1 Heatmap of the median expression of the specific genes for each tissue, shows the degree of specificity.

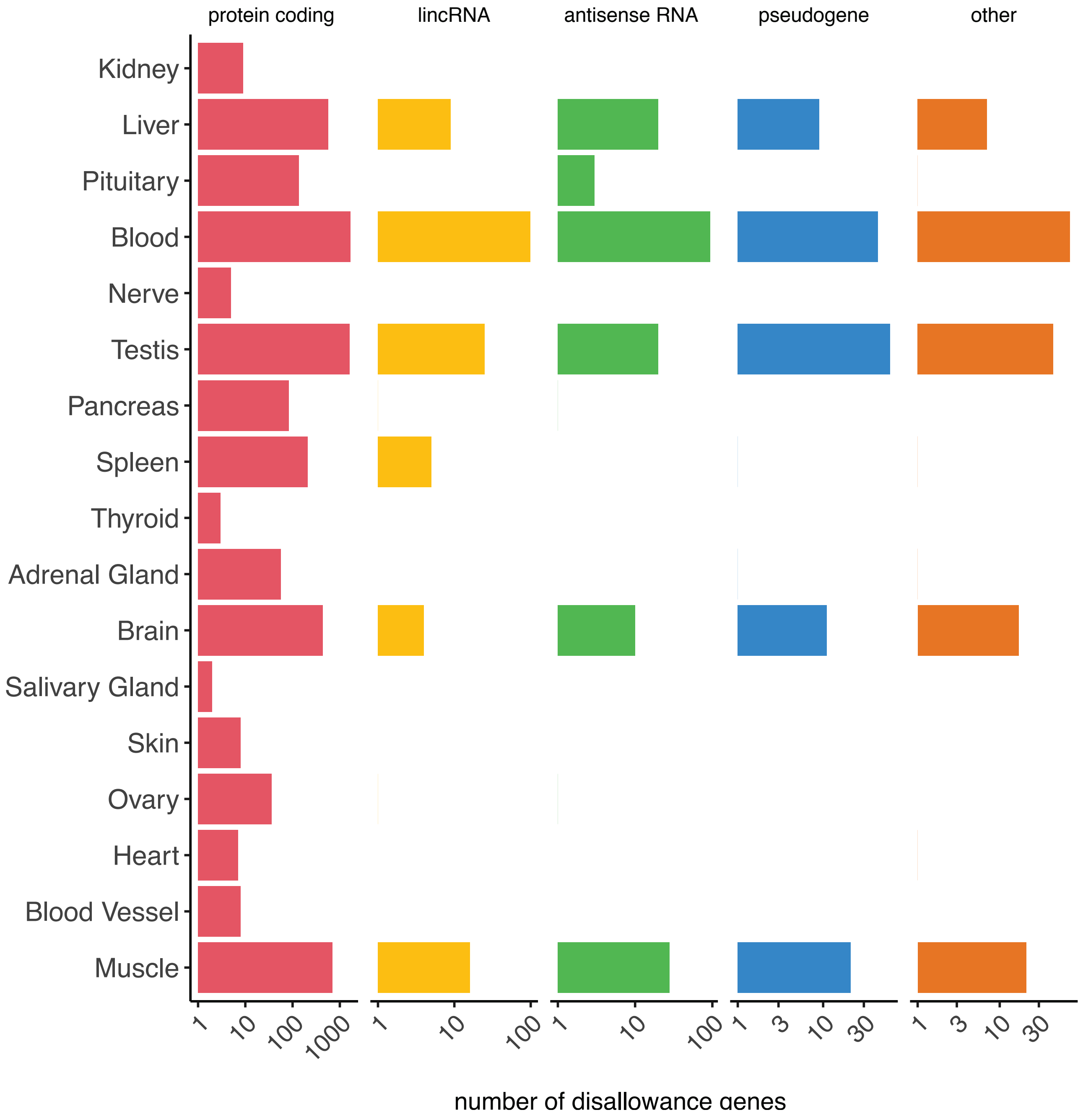

Supplemental Figure 2 Number of disallowance genes for each tissue and biotype is variable.

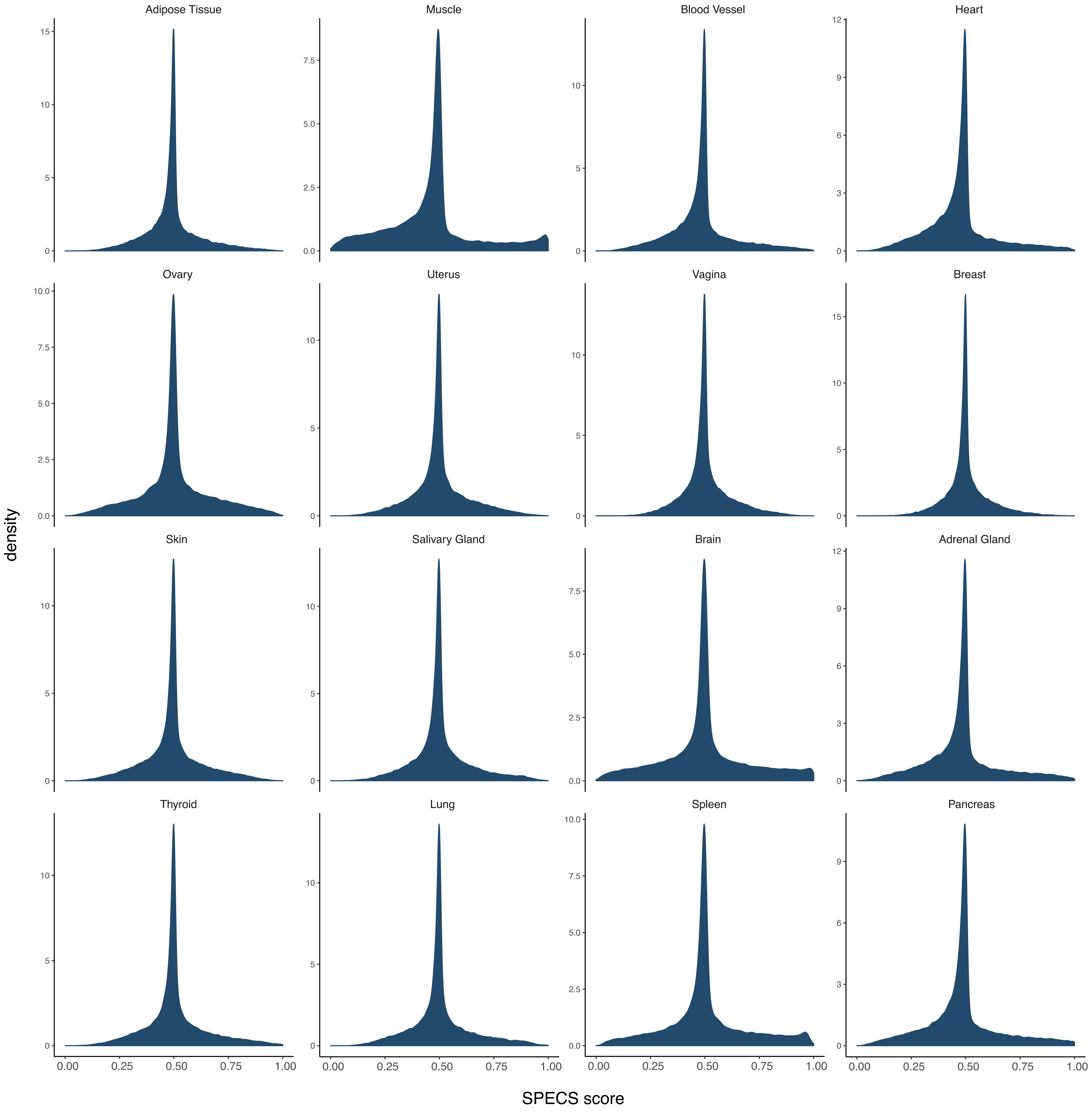

Supplemental Figure 3 SPECS score distribuitions for all GTEx tissue types

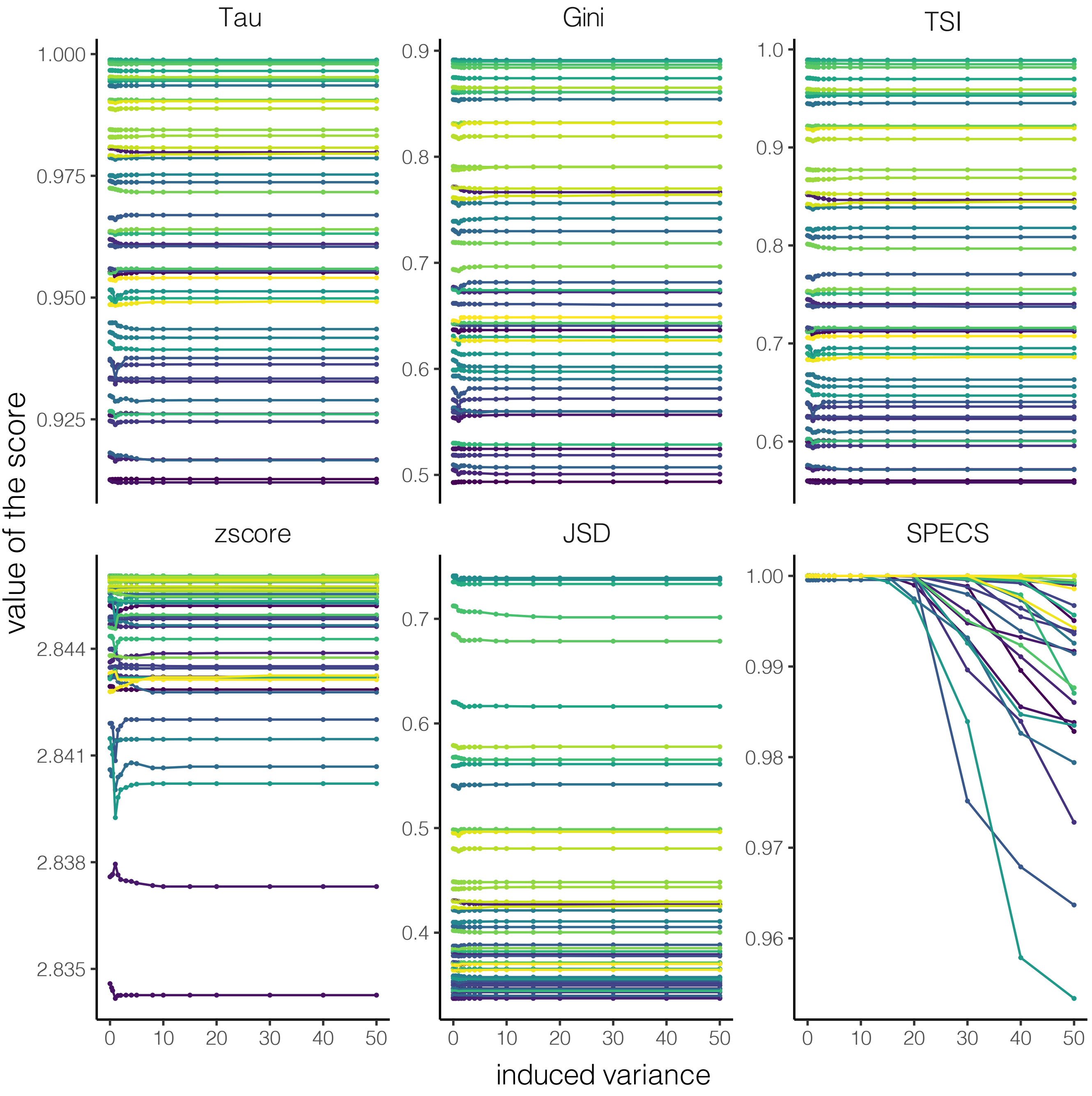

Supplemental Figure 4 Most scores remain stable with a larger induced variance, while SPECS has a declining score
